# Antibacterial Activity of Synthetic Peptides Derived from Lactoferricin against *Escherichia coli* ATCC 25922 and *Enterococcus faecalis* ATCC 29212

**DOI:** 10.1155/2015/453826

**Published:** 2015-03-01

**Authors:** María A. León-Calvijo, Aura L. Leal-Castro, Giovanni A. Almanzar-Reina, Jaiver E. Rosas-Pérez, Javier E. García-Castañeda, Zuly J. Rivera-Monroy

**Affiliations:** ^1^Sciences Faculty, Universidad Nacional de Colombia, Carrera 45, No. 26-85, Bogotá, Colombia; ^2^Medicine Faculty, Universidad Nacional de Colombia, Carrera 45, No. 26-85, Bogotá, Colombia; ^3^University Children's Hospital, University of Würzburg, 97080 Würzburg, Germany

## Abstract

Peptides derived from human and bovine lactoferricin were designed, synthesized, purified, and characterized using RP-HPLC and MALDI-TOF-MS. Specific changes in the sequences were designed as (i) the incorporation of unnatural amino acids in the sequence, the (ii) reduction or (iii) elongation of the peptide chain length, and (iv) synthesis of molecules with different number of branches containing the same sequence. For each peptide, the antibacterial activity against *Escherichia coli* ATCC 25922 and *Enterococcus faecalis* ATCC 29212 was evaluated. Our results showed that Peptides I.2 (RWQWRWQWR) and I.4 ((RRWQWR)_4_K_2_ *Ahx*_2_C_2_) exhibit bigger or similar activity against *E. coli* (MIC 4–33 *μ*M) and *E. faecalis* (MIC 10–33 *μ*M) when they were compared with lactoferricin protein (LF) and some of its derivate peptides as II.1 (FKCRRWQWRMKKLGA) and IV.1 (FKCRRWQWRMKKLGAPSITCVRRAE). It should be pointed out that Peptides I.2 and I.4, containing the RWQWR motif, are short and easy to synthesize; our results demonstrate that it is possible to design and obtain synthetic peptides that exhibit enhanced antibacterial activity using a methodology that is fast and low-cost and that allows obtaining products with a high degree of purity and high yield.

## 1. Introduction

The World Health Organization has stated that control and/or treatment of infections caused by bacteria resistant to conventional drugs is considered a public health goal [1]. Indiscriminate use and inadequate dosage of conventional antibiotics have contributed to the development of resistant bacterial strains, decreasing the therapeutic options [1]. Over the last few decades, several investigations have addressed the development of drugs that do not induce resistance in pathogens and can thus be considered an alternative for the treatment of bacterial infections. Antimicrobial peptides (AMPs) have received special attention as a possible alternative way to combat infections caused by antibiotic-resistant bacterial strains. AMPs are considered to be an important part of the innate immune response, and they have been isolated from tissues and organisms from every kingdom and phylum and have been characterized [2–4]. AMPs have the following characteristics: they are (i) positively charged, (ii) amphipathic, (iii) structurally diverse, and (iv) of short length. AMPs have exhibited antimicrobial activity against Gram-positive and Gram-negative bacteria, fungi, viruses, and parasites [4]. Additionally, AMPs exhibit antibacterial activity over a broad range of pH and temperatures. Interestingly, AMPs have been identified in body fluid proteins in mammals [5], specifically lactoferrin (LF), an 80 kDa non-heme iron-binding protein that is located in mucosal secretions such as breast milk, saliva, seminal plasma, and vaginal mucus [2, 3, 6]. This protein has been associated with biological activities such as antihypertensive, immunomodulator, antitumor, anti-inflammatory, transcription factor, procoagulant, and protease inhibitor activities, among others [7]. Additionally, it has been reported that LF exhibits antimicrobial activity against pathogenic bacteria, fungi, protozoa, parasites, and viruses [8–11]. It has been suggested that LF activity is due to the N-terminal domain [10–13]. When the LF protein reaches the digestive tract, it is digested by gastric pepsin, and the protein hydrolyzate contains a peptide called lactoferricin (Lfcin), which belongs to the N-terminal region [12, 13]. Lfcin has shown greater antibacterial activity against Gram-negative and Gram-positive bacteria than that shown by the protein itself. Some authors have stated that the LF antibacterial activity is mainly due to the Lfcin peptide [2, 12–16]. Lfcin has been identified in several mammals, such as humans (LfcinH), bovine (LfcinB), goats, horses, and pigs [2]. The LfcinB (^17^FKCRRWQWRMKKLGAPSITCVRRA^41^F) has exhibited greater antibacterial activity than what was exhibited by the LfcinH (^20^GRRRRSVQWCAVSQPEATKCFQ-WQRNMRKVRGPPVSC-IKRDSPIQC^67^I). LfcinB inhibits the growth of a wide range of bacteria, viruses, fungi, and parasites [3, 5, 13, 17–21]. Additionally, LfcinB exhibited cytotoxic activity against cancer cell lines, suggesting that LfcinB could be used as an anticancer agent [22–24].

LfcinB contains aromatic amino acids, such as tryptophan (W) and phenylalanine (F), as well as basic residues (e.g., arginine, R and lysine, K) whose side chains provide a net charge of +8 to the peptide. LfcinB contains two cysteine residues that form an intrachain disulfide bridge so that charged and hydrophobic residues are located at opposite sides, providing amphipathic properties to the peptide. Positively charged residues interact electrostatically with the negative charges of bacterial cell wall lipopolysaccharide (LPS), allowing the peptide to approach the bacterial membrane. Then, LfcinB hydrophobic residues interact with the membrane lipid bilayer, causing its disruption and cell lysis [3, 7]. It has been reported that the RRWQWR sequence is the antimicrobial LfcinB center and is considered the smallest motif that exhibits antibacterial and anticancer activity [24, 25]. AMPs can be obtained through solid phase peptide synthesis (SPPS) quickly and inexpensively, with a high degree of purity and good yields [26]. SPPS is a powerful and versatile tool in the design and development of antibacterial agents, which allows the fast and easy production of peptides carrying non-natural amino acid residues and polyvalent molecules, that is, dimeric, tetrameric, and polymeric peptides of a specified amino acid sequence.

In the present paper, the antibacterial activity of synthetic peptides derived from LfcinB containing specific changes in the amino acid sequence was evaluated. These changes were as follows: (i) non-natural amino acid inclusion at specific positions, (ii) sequence length variation, and (iii) multivalent motif presentation, that is, the dimer and tetramer of the RRWQWR sequence. For the experimental strains,* E. coli* ATCC 25922 and* E. faecalis* ATCC 29212 were selected. Our results show that antibacterial activity is enhanced for peptides containing multiple presentations of the RWQWR motif and for peptides derived from LfcinB and LfcinH that contain specific changes in the amino acid sequence.

## 2. Materials and Methods

### 2.1. Reagents and Materials

Mueller-Hinton, Agar SPC,* E. coli* ATCC 25922, and* E*.* faecalis* ATCC 29212 were obtained from ATCC, USA. Rink amide resin, Fmoc-Arg(Pbf)-OH, Fmoc-Asp(OtBu)-OH, Fmoc-Lys(Boc)-OH, Fmoc-Trp(Boc)-OH, Fmoc-Gln(Trt)-OH, Fmoc-*β*-Ala-OH, Fmoc-Phe-OH, Fmoc-Met-OH, Fmoc-Leu-OH, Fmoc-Gly-OH, Fmoc-Ala-OH, Fmoc-Ile-OH, Fmoc-Glu(OtBu)OH, Fmoc-Thr(tBu)-OH, Fmoc-Ser(tBu)-OH, Fmoc-Val-OH, Fmoc-Pro-OH, Fmoc-Cys(Trt)-OH, Fmoc-Lys(Fmoc)-OH, 6-(Fmoc-amino)hexanoic acid (Fmoc-*Ahx*-OH), 1-hydroxybenzotriazole (HOBt), and* N,N*-dicyclohexylcarbodiimide (DCC) were purchased from AAPPTec (Louisville, KY, USA).* N,N*-Diisopropylethylamine (DIPEA), triisopropylsilane (TIPS), 1,2-Ethanedithiol (EDT), 4-methyl-piperidine, pyridine, ninhydrin, phenol, and KCN were purchased from Sigma-Aldrich (St. Louis, MO, USA). Methanol, diethyl ether,* N,N*-dimethylformamide (DMF), absolute ethanol, dichloromethane (DCM), acetonitrile (ACN), isopropyl alcohol (IPA), and trifluoroacetic acid (TFA) were obtained from Honeywell-Burdick & Jackson (Muskegon, Michigan, USA). All reagents were used without further purification.

### 2.2. Peptide Synthesis

Peptides were synthesized using the SPPS-Fmoc/tBu methodology [27]. Briefly, Rink amide resin (100 mg) was used as solid support. (i) The resin conditioning and Fmoc group removal were carried out through treatment with 20% 4-methyl-piperidine in DMF at room temperature (RT) for 10 minutes twice. Then, the resin was exhaustively washed with DMF, IPA, and DCM. (ii) For the coupling reaction, 0.21 mmol of Fmoc-amino acids was preactivated with DCC/HOBt (0.20/0.21 mmol) in DMF at RT. The activated Fmoc-amino acid was added to a reactor containing deprotected resin; the coupling reaction was shaken for two hours at RT, and then the resin was washed. (iii) Fmoc group elimination and the incorporation of each amino acid were confirmed through the ninhydrin test [28]. Side chain deprotection reactions and peptide separation from the resin were carried out with a cleavage cocktail containing TFA/water/TIPS/EDT (93/2/2.5/2.5% v/v). The cleavage mixture was filtered and the solution was collected. Crude peptides were precipitated via treatment of the solution with cool ethyl ether, and finally the products were washed with ether 5 times and dried.

### 2.3. Analytical Methods

Reverse phase HPLC (RP-HPLC) analysis was performed on an Agilent Eclipse XDB-C18 (4.6 × 150 mm, 3.5 *μ*m) column using an Agilent 1200 liquid chromatograph (Omaha, Nebraska, USA). For the analysis of crude peptides (20 *μ*L, 1 mg/mL), a linear gradient was applied from 5% to 70% Solvent B (0.05% TFA in ACN) in Solvent A (0.05% TFA in water) for 45 min at a flow rate of 1.0 mL/min at RT and 210 nm detection. The crude products were purified through solid-phase extraction (SPE), using Supelclean LC-18 SPE columns that were activated and equilibrated prior to use. Crude peptides were passed through the column, and a gradient was used for their elution [29]. Collected fractions were analyzed using RP-HPLC (as describe above) and MS. MALDI-TOF MS analysis was performed on an Ultraflex III TOF-TOF mass spectrometer (Bruker Daltonics, Bremen, Germany) in reflectron mode, using an MTP384 polished steel target (Bruker Daltonics), 2,5-dihydroxybenzoic acid, or sinapinic acid as a matrix, 500 shots with 25–30% power laser.

### 2.4. Susceptibility Testing

The bacterial strain* E. coli* ATCC 25922 was grown in Mueller Hinton broth (MH) from 18 to 24 hours at 37°C in an aerobic atmosphere. CFU/mL was calculated, and the inoculum was diluted to a 1 × 10^6^ CFU/mL concentration. An aliquot was placed on MH agar plates, mixed, and allowed to solidify. Five wells were drilled using a punch of 8 mm, and then each hollow was filled with 100 *μ*L of peptide (2000 *μ*g/mL). Incubation for 24 hours at 37°C was then performed.

### 2.5. Antibacterial Activity

The minimum inhibitory concentration (MIC) and minimum bactericidal concentration (MBC) were determined using the microdilution assay [30]. Briefly, bacterial strains were incubated for 18 to 24 hours at 37°C in MH broth until an optical density of 0.15 to 0.30 (620 nm) was obtained. Using a 96-well microtiter plate, peptide serial dilution (200, 100, 50, 25, 12.5. 6.2 *μ*g/mL) was performed, and then they were incubated for 24 h at 37°C, with an inoculum of 2 × 10^6^ CFU/mL in MH broth. The final volume in each well was 100 *μ*L. After incubation for 18 h, the absorbance at 620 nm was measured using an Asys Expert Plus ELISA reader. For determining the MBC, using an inoculation loop, a small sample was taken from each well and then was spread on MH agar plates and incubated overnight at 37°C (*n* = 2).

## 3. Results

Peptides derived from LfcinB and LfcinH proteins were designed (Table 1) and synthesized through SPPS using the Fmoc/tBu strategy. The crude products were characterized using RP-HPLC and then purified via SPE chromatography. In all cases, chromatographic profile of the purified products exhibited a mainly specie. MALDI-TOF-MS analysis showed that synthesized peptides had the expected molecular weight. Table 1 presents a summary of the RP-HPLC and MALDI-TOF-MS analysis.

Designed peptides were organized in four groups as follows: Group I and Group II, peptides containing the sequence RWQWR. The peptides in these groups were designed to establish if the antimicrobial activity could be affected by the introduction of non-natural amino acids, amino acid substitutions, truncated sequences, and/or multiple motif presentation, that is, palindromic or tetrameric sequence. Group III corresponds to sequences derived from N-terminal region of LfcinH. Finally, controls (Group IV) comprised the LFB protein, LfcinB synthetic peptide (Peptide IV.1), and a nonrelevant sequence PrM protein belonging to Dengue virus (Peptide IV.3).

**Figure 1:**
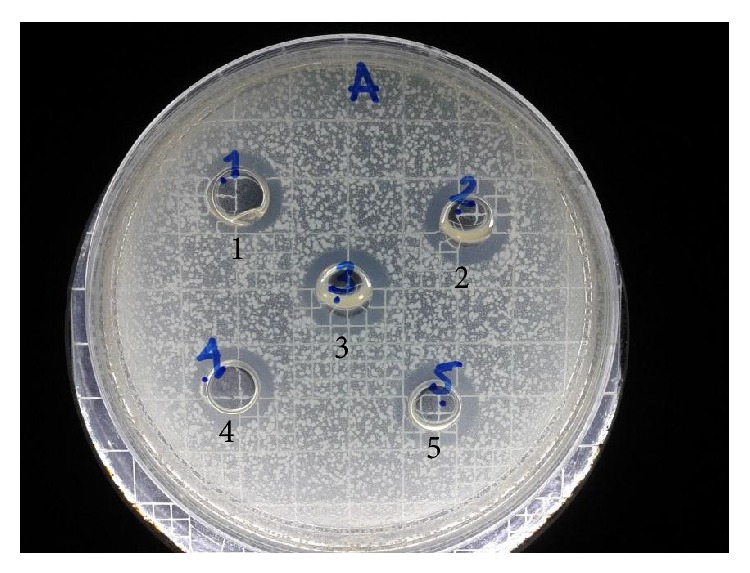
Susceptibility assays against* E. coli* ATCC 25922. Peptide II.8 (1), Peptide II.4 (2), Peptide II.3 (3), Peptide I.3 (4), and Peptide II.5 (5).

Susceptibility assays were performed to determine if the designed peptides exhibited antibacterial activity against the selected strains. All peptides showed an inhibition zone ranging from 12 to 14 mm, indicating that these peptides can inhibit bacterial growth (Figure 1). Significant differences in the size of the inhibition zone caused by the tested peptides were not found. This could be due to the high concentration (2000 *μ*g/mL) used. Then optimal conditions were established to determine the MIC and MBC for each peptide against* E. coli* and* E. faecalis* (Table 1).

## 4. Discussion

### 4.1. Antibacterial Activity of Lactoferricin-Derivated Peptides against* E. coli* ATCC 25922

**Figure 2:**
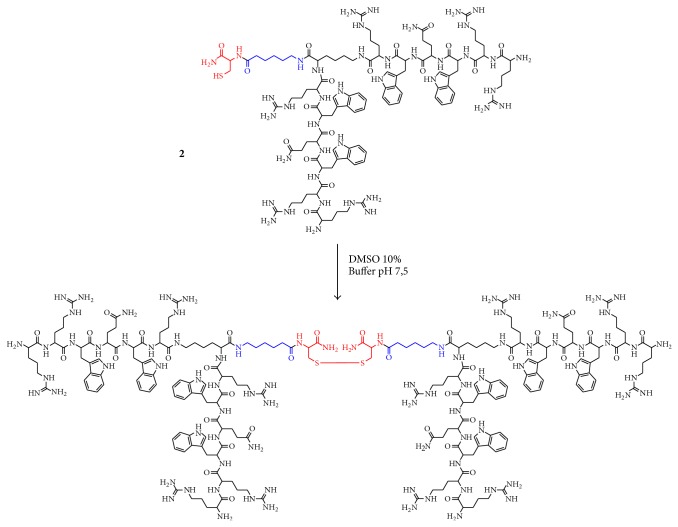
Synthesis of Peptide I.4. A dimer (top) was first synthesized and purified; this molecule contains two copies of the sequence RRWQWR, a spacer (*Ahx*, in blue), and a cysteine residue (in red). The tetra branched peptide (bottom) was obtained by oxidation of dimer molecule.

MIC and MBC values obtained against* E. coli* ATCC 25922 showed that Peptides I.2 and I.4 (Table 1) have the highest antibacterial activity against this strain, MIC 4 and 27 *μ*M, respectively. Peptide I.4 corresponds to a branched peptide that contains 4 copies of the RRWQWR motif; it showed greater antibacterial activity than the sequence RRWQWR itself (Peptide I) and the controls, synthetic LfcinB (Peptide IV.1), and native protein (IV.2). This result indicates that multiple copies of the RRWQWR sequence could enhance the antibacterial activity. Peptide I has been considered as the minimum motif with antibacterial action, and its activity has been attributed to the presence of Trp and Arg residues in an alternating way. These amino acids have been considered important in the mechanism of antibacterial activity of LfcinB [31–33]. For* E. coli*, the palindromic sequence RWQWRWQWR (Peptide I.2) exhibited greater antibacterial activity than that showed by Peptide I and was similar to the controls (IV.1 and IV.2). This palindromic sequence contains the motif WQW, flanked by Arg residues, conferring amphipathic characteristics to peptides that have been considered as relevant in the action mechanism proposed for Lfcin. When a beta-alanine residue was introduced at the N-terminal end (Peptide I.3), the antibacterial activity was reduced. This result suggests that positive charge density over the Arg residue at the N-terminal is relevant to the activity of this peptide, probably because of electrostatic interaction with the bacterial membrane. Our results indicate that antibacterial activity was increased with the multiplicity of motif RRWQWR and are in agreement with a previous report, where it was demonstrated that MAPs (multiple antigen peptides) of a sequence derived from LfcinH have significant antibacterial activity [34]. However, the synthesis of a sixteen-branched peptide is a high-cost process that gives low yields and is time consuming, due principally to steric hindrance. Our synthetic strategy is simpler because a two-branched peptide was first synthetized using SPPS-Fmoc/tBu. This reduced the problems related to steric hindrance. This method allowed us to obtain a dimeric peptide carrying a cysteine residue with no major difficulties in a process that is rapid and reproducible, gives high yields, and is of high purity. Purified dimer was oxidized using DMSO to generate the tetra-branched peptide (I.4) through disulfide bond formation (Figure 2). Comparing our results with previous reports of other authors, it was reported that RRWQWR presented a MIC of 15 *μ*M against* E. coli* ML35 [33], whereas in our study this sequence (Peptide I) showed a MIC of 100 *μ*M against* E. coli* ATCC 25922, showing that antibacterial activity of this sequence is dependent on the strain.

For Group II, the highest antibacterial activity against* E. coli* was exhibited by Peptide II.1, followed by Peptides II.2, II.8, and II.4. When the results obtained with Peptides II.4 to II.7 are compared, it was possible to establish that (i) cysteine residue at the 17th position is not relevant to the antibacterial activity; previously, for LfcinB, it was reported that reduction of disulfide bridge does not affect the antibacterial activity [35]; (ii) the replacement of Arg by Leu residues at positions 20 and 21 dramatically reduced the activity (Peptides II.6 and II.7); (iii) a beta-alanine residue at the N-terminal end (Peptides II.8 and II.9) considerably reduced the antibacterial activity, similar to the result discussed above (Peptide I.3). Our results suggest that RRWQWRM corresponds to the minimum sequence that exhibits activity against* E. coli.* When this motif was flanked, the antibacterial activity was affected. Peptide II.1 has been tested by other authors and has received several names (LFB, LFB (17-31), LfcinB 17-31, and LfcinB15). Our results for Peptide II.1 (MIC and MCB 25 *μ*M) are in agreement with those reported by others, that is, MIC 24 *μ*M [36], MIC 24 *μ*M [36, 37], MIC 20 *μ*M [38], MIC 30 *μ*g/mL and MBC 40 *μ*g/mL [39], and MIC 32 *μ*g/mL [32]. In the same way, our Peptide II.8 exhibits an activity (MIC and MBC 32 *μ*M (50 *μ*g/mL)) similar to that reported by others, that is, MIC/MBC 32/128 *μ*g/mL [32], 50/50 *μ*g/mL [40] and MIC 50 *μ*M [41].

The antibacterial activity obtained for Peptide III.1 that corresponds to LfcinH (20-30) was MIC 18 *μ*M. Modification of this sequence by the incorporation of a beta-alanine (*β*A) residue at the N-terminal end does not change the activity (Peptide III.4, MIC 17 *μ*M). However, when the *β*A was introduced at the C-terminal end, the activity was reduced significantly (Table 1. III.2, III.3). Peptide III.1 is known as hLF (1-11), and it has exhibited antibacterial activity against* E. coli* O54 and has reduced the number of viable bacteria in mice infected with resistant strains of* S. aureus* and* K. pneumonia*. The authors stated that Arg residues at the N-terminal end (^21^R and ^22^R) are relevant to the antibacterial activity of this sequence [42].

The antibacterial activity of Peptide IV.1 was similar to that of the native protein LF (control IV.2). The results for synthetic Peptide IV.1 (MIC and MBC 32 *μ*M, corresponding to 100 *μ*g/mL) against* E. coli* ATCC 25922 are in agreement with the results reported by other authors for the same synthetic peptide (MIC/MBC 30/80 *μ*g/mL [39] and MIC 30 *μ*g/mL [38]). Interestingly, it has been reported that LficnB, obtained by protein hydrolysis, presents higher antibacterial activity: MIC 6 *μ*g/mL (*E. coli* O111), MIC 6 *μ*g/mL (*E. coli* IID861) [43], MIC 50 *μ*g/mL (*E. coli* IID861) [44], MIC 32 *μ*g/mL (*E. coli* ATCC 25922), and MIC 64 *μ*g/mL (*E. coli* K88) [32].

### 4.2. Antibacterial Activity of Lactoferricin-Derivated Peptides against* E. faecalis* ATCC 29212

The antibacterial activity results for Peptides I.4, I.2, and II.1 against* E. faecalis* were similar to those established for* E. coli*; that is, in the same way as for* E. coli*, Peptide I.4 (tetramer peptide) exhibits the best antibacterial activity against this strain, showing a smaller MIC than Peptide II.1 and the native protein itself. Interestingly, and in contrast to* E. coli*, the inclusion of beta-alanine residue at the N-terminal end does not affect the antibacterial activity against* E. faecalis* (Peptides I.2 and I.3). Additionally, Peptide II.10 shows good activity against this strain (Table 1). Please note that, for this peptide, two lysine residues (K) were replaced by arginine residues (R), suggesting that not only the charge but also its nature is significant and relevant to the activity. For Group II, it is important to note that most of the specific changes performed in the Peptide II.1 sequence reduced the antibacterial activity against* E. faecalis* ATCC 29212. The synthetic LfcinB and the LF native protein exhibit antibacterial activity against* E. faecalis* ATCC 29212 (Table 1). These results contrast with those obtained by Bellamy et al. [43], who reported that the* E. faecalis* ATCC E19433 strain was resistant to all evaluated concentrations of LfcinB. That group had obtained LfcinB by hydrolysis of lactoferrin using pepsin. On the other hand, our results are in agreement with the analysis presented by Chen et al. [45], which demonstrated that peptides containing Arg- and Trp-rich sequences exhibited a high degree of antibacterial activity against* E. faecalis* ATCC 29212. It is also interesting to note that peptides from Group III did not show a high degree of antibacterial activity against* E. faecalis*.

The results obtained for both strains can be summarized as follows: (i) three sequences (Peptides I.2, I.4, and II.1) exhibited a high degree of antibacterial activity against* E. coli* and* E. faecalis*, suggesting that these peptides may present a broad spectrum of antibacterial activity. Some peptides exhibited antibacterial activity against a specific strain; thus, (ii) Peptides II.2, II.4, II.8, III.1, and III.4 only exhibited activity against* E. coli*, and (iii) Peptide II.10 exhibited activity specifically against* E. faecalis*. We studied the influence, for antibacterial activity, of introducing specific changes to peptide sequences from bovine lactoferricin, such as (i) incorporation of non-natural amino acids, (ii) reduction or elongation of the motif, (iii) replacement of basic residues by noncharged residues, and (iv) multiple presentations of the RWQWR motif, such as a tetra-branched or palindromic sequence. We found that these changes directly influenced the antimicrobial activity. The types of microorganisms and their characteristics also affected the action of each peptide. The behavior of the antibacterial activity with the changes in the sequences did not follow a characteristic pattern; its behavior was specific to each microorganism. Our results suggested that peptide antibacterial activity is probably dependent on bacteria and/or the bacterial strain evaluated. This is in agreement with the results found by other authors [2, 32, 40, 46] who had reported that antibacterial activity of peptides derived from LfcinB was dependent on both the kind of bacteria and its strain.

## Figures and Tables

**Table 1:** Synthetic peptides derived from lactoferricin protein. Summary of characterization (RP-HPLC and MALDI-TOF MS) and antibacterial activity of purified products.

Group	Peptide code	Sequence	RP-HPLC *t*_*R*_ (min)	MALDI-TOF MS		*E. coli* ATCC 25922		*E. faecalis* ATCC 29212	
				*Theoretical* Mol wt	*Experimental* *m/z*. [M + H]^+^	MIC (*µ*M)	MCB (*µ*M)	MIC (*µ*M)	MCB (*µ*M)
I	I	RRWQWR	16,73	985,54	986,92	101,5	101,5	202,9	202,9
	I.1	RRWQWR βA	16,28	1057,56	1057,27	94,6	189,1	189,1	189,1
	I.2	RWQWRWQWR	22,84	1485,75	1487,12	26,9	33,7	26,9	33,7
	I.3	βA RWQWRWQWR	22,47	1556,79	1558,16	64,2	64,2	32,1	32,1
	I.4	((RRWQWR)_2_KAhxC)_2_	20,00	4596,64	2300,57^a^	4,4	4,4	10,9	10,9

II	II.1	^ 17^FK^19^ **C** RRWQWRMKKLG^31^A	19,30	1993,49	1995,02	25,1	25,1	25,1	25,1
	II.2	FKβA RRWQWRMKK	17,42	1718,97	1721,12	29,1	29,1	116,3	116,3
	II.3	FK A RRWQWRMKK	17,81	1718,97	1720,81	116,3	116,3	58,2	116,3
	II.4	FK A RRWQWRM	19,51	1462,78	1464,73	34,2	34,2	68,4	136,7
	II.5	FKβA RRWQWRM	19,17	1462,78	1464,51	68,4	136,7	136,7	136,7
	II.6	FK ARL WQWRM	20,13	1420,75	1420,93	70,4	70,4	140,8	140,8
	II.7	FK ALL WQWRM	23,14	1377,72	1377,9	145,2	145,2	145,2	145,2
	II.8	RRWQWRMKKLG	18,31	1542,87	1545,12	32,4	32,4	64,8	129,6
	II.9	βA RRWQWRMKKLG	18,19	1613,91	1615,3	123,9	123,9	62,0	123,9
	II.10	RRWQWRMRRLGβA	18,52	1669,92	1672,64	59,9	59,9	15,0	29,9
	II.11	RRWQWRMKKβA	17,17	1443,80	1445,08	69,3	69,3	69,3	69,3

III	III.1	^ 20^GRRRRSVQWC^30^A	16,00	1372,73	1373,6	18,2	36,4	72,8	145,7
	III.2	βAGRRRRSVQWCAβA	14,81	1516,76	1515,98	65,9	65,9	65,9	131,9
	III.3	GRRRRSVQWCAβA	15,15	1445,68	1144,93	69,2	138,3	69,2	138,3
	III.4	βAGRRRRSVQWCA	15,20	1443,76	1444,82	17,3	34,6	69,3	138,5

IV	IV.1	FKCRRWQWRMKKLGAPSITCVRRAE	19,12	3104,66	3106,15	32,2	32,2	32,2	32,2
	IV.2	LF protein	—	80000	—	25,0	25,0	25,0	25,0
	IV.3	ITEVEPEDIDT	15,02	1258,58	1259,99	1589,1	1589,1	1589,1	1589,1

^a^This *m*/*z* signal corresponds to the dimer before oxidation (see Figure 2). The reported antimicrobial LfcinB center [24, 25] is underlined and changes in amino acid sequences are in box.
